# Hepatic Stellate Cells Secreted Hepatocyte Growth Factor Contributes to the Chemoresistance of Hepatocellular Carcinoma

**DOI:** 10.1371/journal.pone.0073312

**Published:** 2013-09-02

**Authors:** Guofeng Yu, Yingying Jing, Xingrui Kou, Fei Ye, Lu Gao, Qingmin Fan, Yang Yang, Qiudong Zhao, Rong Li, Mengchao Wu, Lixin Wei

**Affiliations:** 1 Tumor Immunology and Gene Therapy Center, Eastern Hepatobiliary Surgery Hospital, the Second Military Medical University, Shanghai, China; 2 Department of Comprehensive Treatment, Eastern Hepatobiliary Surgery Hospital, the Second Military Medical University, Shanghai, China; 3 Soochow University, Suzhou, Jiangsu, China; The University of Hong Kong, China

## Abstract

As the main source of extracellular matrix proteins in tumor stroma, hepatic stellate cells (HSCs) have a great impact on biological behaviors of hepatocellular carcinoma (HCC). In the present study, we have investigated a mechanism whereby HSCs modulate the chemoresistance of hepatoma cells. We used human HSC line lx-2 and chemotherapeutic agent cisplatin to investigate their effects on human HCC cell line Hep3B. The results showed that cisplatin resistance in Hep3B cells was enhanced with LX-2 CM (cultured medium) exposure in vitro as well as co-injection with LX-2 cells in null mice. Meanwhile, in presence of LX-2 CM, Hep3B cells underwent epithelial to mesenchymal transition (EMT) and upregulation of cancer stem cell (CSC) -like properties. Besides, LX-2 cells synthesized and secreted hepatic growth factor (HGF) into the CM. HGF receptor tyrosine kinase mesenchymal–epithelial transition factor (Met) was activated in Hep3B cells after LX-2 CM exposure. The HGF level of LX-2 CM could be effectively reduced by using HGF neutralizing antibody. Furthermore, depletion of HGF in LX-2 CM abolished its effects on activation of Met as well as promotion of the EMT, CSC-like features and cisplatin resistance in Hep3B cells. Collectively, secreting HGF into tumor milieu, HSCs may decrease hepatoma cells sensitization to chemotherapeutic agents by promoting EMT and CSC-like features via HGF/Met signaling.

## Introduction

Hepatocellular carcinoma (HCC) which accounting for 70% to 85% of the total primary liver cancer, is one of the most common primary malignant tumors with a fairly high and increasing incidence, frequently relapse and dismal prognosis [1]. Nowadays, radical surgery is considered as the first line of therapy for HCC, while systematic chemotherapy plays a pivotal role in eradicating the microscopic residual and curing the patients who are not feasible for surgery [2]. However, response rate and effect on overall survival as the result of chemotherapy are exceedingly limited because of the inevitable generation of drug resistance in the course of chemotherapy treatment [3]. The intimate cross-talk between tumor cells and their surrounding microenvironment plays an extremely important role in modulating the biological behaviors of tumor, and eventually affects clinical outcome [4].

Cancer cells are in contact with their surrounding cells by paracrine and autocrine mechanisms. The abbrent networks of growth factors, cytokines, chemokines and their cognate receptors, are tightly involved in cancer progression [5]. Hepatic stellate cells (HSCs) are protean, multifunctional, and enigmatic cells of the liver. In HCC, HSCs secrete soluble cytokines, chemokines, chemotaxis to create the complexity of tumor milieu [6]. Recent reports have confirmed that reciprocal signaling between HSCs and precancerous hepatocytes or hepatoma cells facilitates tumorigenesis, migration, invasion, metastasis formation [5,7]. Additionally, tumor microenvironment heterogeneity and plasticity may lead to the diversities of cell proliferation rate, significant regional gradient of hypoxia and acidic zone, all of which would influence the sensitivity of tumor cells to chemotherapy [8]. Moreover, tumor stroma is also involved in mediating epithelial to mesenchymal transition (EMT) and maintaining the cancer stem cell (CSC) -like characteristics of epithelial tumor cells, both of which are responsible for cancer chemoresistance [9,10].

Based on these premises, the purpose of this study was to understand the role of HSCs in mediating the chemoresistance phenotype of hepatoma cells and the mechanism by which this occurs. Simultaneously, considering the crucial effects of EMT and CSC in cancer chemoresistance, we also explored if tumor cells underwent EMT and upregulation of CSC phenotypes under the role of HSCs in our experiments.

## Materials and Methods

### Xenografts in Nude Mice

The animal experiments were approved by the Clinical Research Ethics Committee of Eastern Hepatobiliary Surgery Hospital. Male athymic (BALB/c-nu/nu) mice (6-week-old) were purchased from Shanghai Experimental Animal Center, Chinese academy of science. All animals were maintained in a pathogen-free environment and given radiation-sterilized food pellets and distilled water. Aged 8 to 12 weeks of animals were used and all experiments were performed following the Laboratory Animal Centre care guidelines and mice were sacrificed by cervical dislocation. BALB/c nu/nu mice were randomized into four groups: control, LX-2, cisplatin and LX-2 plus cisplatin group, each group was consisted of 4 animals. The armpit of mice from the control and cisplatin group was injected subcutaneously with 4×10^5^ of exponentially growing Hep3B cells suspended in 100µl PBS; The armpit of mice from the LX-2 and LX-2 plus cisplatin group was injected with the mixture of 4×10^5^ of exponentially growing Hep3B cells and 2×10^5^ of exponentially growing LX-2 cells suspended in 100µl PBS. When tumors reached a volume between 100 and 150 mm^3^ about in two weeks, cisplatin (1mg/kg, diluted in 50µl of 0.9% saline, Qilu Pharmaceutical Co., Ltd.) was intratumoral injection of mice from cisplatin and LX-2 plus cisplatin group every other day by using insulin syringes. Correspondingly, equal volume of 0.9% saline was intratumoral injection of mice from control and LX-2 group every other day [11]. Mice were sacrificed by cervical dislocation at the 30th day. Tumors at the inoculation site were removed. Tumor volume and mass weight were measured.

### Cell culture

The human HCC cell line Hep3B was supported with Dulbecco’s Modified Eagle Medium (DMEM, Gibco-BRL, Gaithersburg, MD, USA) containing 10% FBS (Fetal bovine serum) and antibiotics (100mg/L penicillin and 100mg/L streptomycin); human HSC cell line LX-2 was maintained in RPMI 1640 (Gibco-BRL, Gaithersburg, MD, USA) supplemented with 10% FBS and antibiotics (100mg/L penicillin and 100mg/L streptomycin). Both cells were incubated in a humidified thermostat under 5% CO2 at 37^o^C.

### Conditioned medium and ELISA

Cultured media (CM) were collected from LX-2 cells cultured in 2D culture dishes and centrifuged at 1000g for 5 minutes to obtain the supernatant. LX-2 CM was incubated with HGF antibody (GTX10678, 0.5 µg/ml) for 24h at 4^o^C to obtain HGF-depleted LX-2 CM. The level of HGF and TGF-β in CM waere determined by using human HGF ELISA kit (HY10158E, Shanghai HengYuan Biological Technology Co., Ltd) and TGF-β ELISA kit (HY10108E, Shanghai HengYuan Biological Technology Co., Ltd) according to manufacturer’s instructions.

### Cell viability assay

Hep3B cells (5×10^3^ cells/well) in the exponential phase of growth were seeded into a 96-well tissue culture plate and incubated for 24 h. The cells were treated with cisplatin (10 µg/ml) for 12, 24 and 48 h to be tested. The cytotoxicity of cisplatin was determined by cell counting Kit-8 (CCK-8) assay according to the manufacturer’s instructions.

### Apoptosis assay

Cisplatin induced apoptosis of Hep3B cells was detected by flow cytometry using Annexin V-FITC Apoptosis Detection Kit (KGA, KeyGEN, Biotech) according to the manufacturer’s instructions. Briefly speaking, the pretreated cells were harvested and stained with PI and Annexin V-FITC. The apoptosis rate was assayed by using FACSCalibur Flow Cytometry (Becton Dickinson and Beckman-Coulter, San Jose, CA, USA) at 488nm.

### Real time PCR

The total RNA from Hep3B cells was extracted by Trizol (Invitrogen, Carlsbad, CA) according to the manufacturer’s protocol. Real time PCR was performed with stepOne Real-time PCR systems at the condition of 95°C for 10 min, followed by 40 cycles of 95°C for 15 s, 60°C for 30s and 72°C for 30s and ended with 95°C for 1 min, 55°C for 30s, 95°C for 30s. The primers used were listed in Table 1.

**Table 1:** The primers were used in the experiments.

E-cadherin	Forword	5’-TCAGCCGCTTTCAGATTTTCA-3’
	Reverse	5’-ATGAGTGTCCCCCGGTATCTT-3’
N-cadherin	Forword	5’-TGGATGGACCTTATGTTGCT-3’
	Reverse	5’- AACACCTGTCTTGGGATCAA-3’
Vimentin	Forword	5’-GCAATCTTTCAGACAGGATGTTGAC-3’
	Reverse	5’-GATTTCCTCTTCGTGGAGTTTCTTC-3’
Bmi1	Forword	5’-ACAGTCTCAGGTATCAACCAG-3’
	Reverse	5’-CCAGCGGTAACCACCAATC-3’
Klf4	Forword	5’-GTCGGACCACCTCGCCTTACACAT-3’
	Reverse	5’-GGTCTTCCCTCCCCCAACTCACG-3’
GAPDH	Forword	5’-CCACATCGCTCAGACACCAT-3’
	Reverse	5’-GCGCCCAATACGACCAAAT-3’

### Western-blot

Protein samples were acquired directly by cell extraction buffer (Beyotime, P0013) containing a protease inhibitor PMSF (Cwbiotech, CW0037). The equivalent aliquots of proteins were electrophoresed on a 10% SDS/polyacrylamide gel in 1XTris-glycin buffer and transferred to nitrocellulose membranes. Nonspecific binding was blocked by incubation with 5% nonfat milk for 2 hours before overnight incubation with primary antibodies against E-cadherin (ab53033, diluted 1:1000), N-cadherin (ab18620, diluted 1:500), Vimentin (ab135708, diluted 1:2000), active Caspase 8 (CST, #9748, diluted 1:1000), active Caspase 3 (CST, #9664, diluted 1:500), Bmi1 (ab126783, diluted 1:1000), Klf4 (ab26648, diluted 1:2000), Met (CST, #8041, diluted 1:1000), p-Met (CST, #3126, diluted 1:1000) and GAPDH (ab9385, diluted 1:5000) as control. Following incubated with secondary antibody (horseradish peroxidase-conjugated rabbit or mouse anti-human IgG, ab6715, diluted 1:10000), the immunoreactive proteins were detected by enhanced chemoluminescence substrate, and the blot was scanned and densitometric analysis with ImageJ software.

### Flow cytometry analysis of CD133

The expression of surface marker of CSC, CD133 was analyzed with FACSCalibur Flow cytometry (Becton Dickinson and Beckman-Coulter, San Jose, CA, USA) according to manufacturer’s instructions. Cells after pretreatment were trypsinized, centrifuged, suspended with PBS and incubated with FcR blocking reagent (Miltenyi Biotec, Germany), and then stained with the directly conjugated monoclonal antibody, anti-human CD133-PE (Miltenyi Biotec, Germany). Flow analysis was performed on FACSCalibur Flow Cytometry.

### Immunofluorescence

Pretreatment cells were fixed with 4% paraformaldehyde and incubated with β-catenin (diluted 1:200) or Vimentin (diluted 1:200) overnight at 4^o^C and then appropriate Alexa Fluor 488-labeled secondary antibodies (Molecular Probes, Invitrogen, Paisley, UK) for 30 minutes at room temperature. Nuclei were counterstained with DAPI (Sigma-Aldrich). The cells were subsequently scanned with a confocal microscope (Leica TCS SP2).

### Statistical analysis

Data were shown as mean ± standard error. Differences between groups were performed by one-way ANOVA and Tukey’s multiple comparison test. P value lower than 0.05 was considered statistically significant.

## Results

### Enhancement of cisplatin resistance of Hep3B cells by LX-2 cells

**Figure 1:**
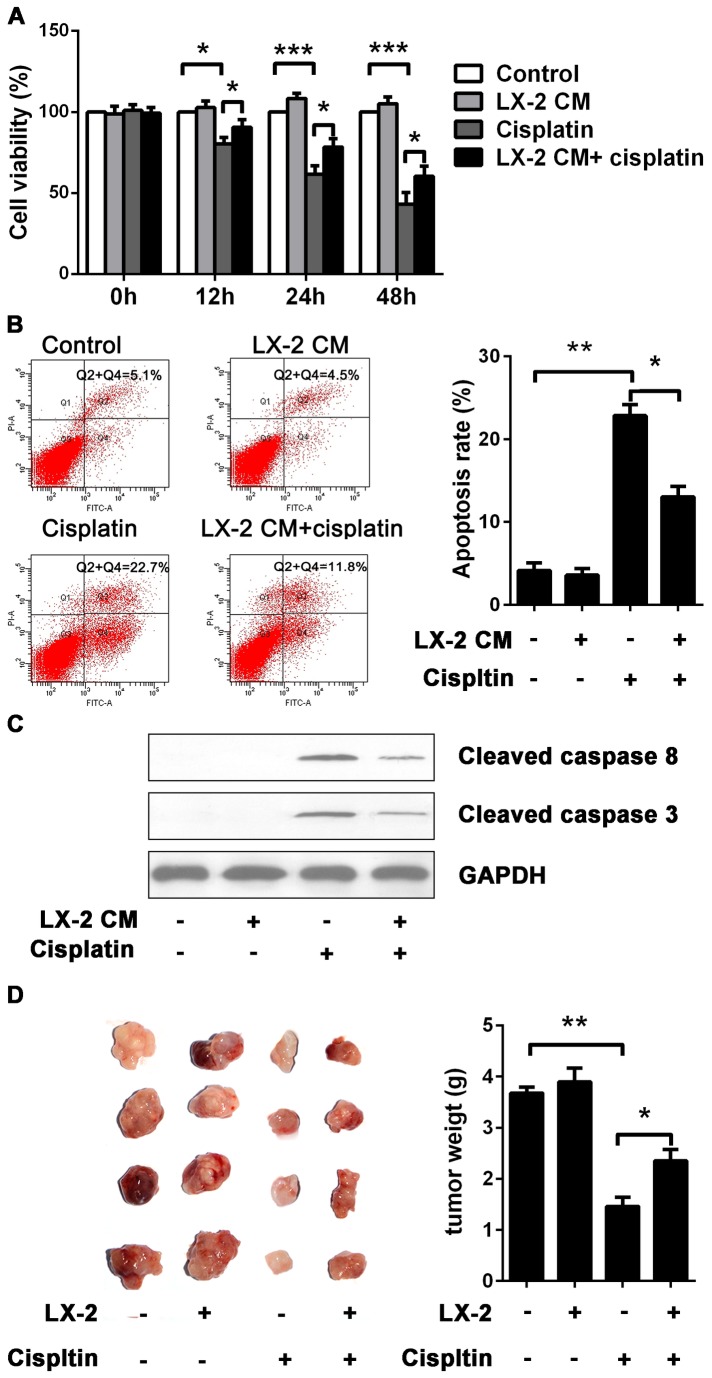
Induction of cisplatin resistance in Hep3B cells by LX-2 cells. (A) The effect of LX-2 CM on cisplatin-induced cell proliferation arrest. Hep3B cells were treated with cisplatin at the concentration of 10 µg/ml for 12, 24 and 48 h with or without LX-2 CM exposure. Cell viability was measured by using a CCK8 assay. Compared to Hep3B cells cultured in normal medium, cisplatin induced cytotoxicity was markedly attenuated in Hep3B cells upon LX-2 CM exposure. (B) Anti-apoptotic effect of LX-2 CM on cisplatin-induced apoptosis. Hep3B cells with or without LX-2 CM exposure were treated with cisplatin at the concentration of 10 µg/ml for 24h. The apoptosis rate was determined by using flow cytometry with Annexin V-FITC and PI double staining. In response to cisplatin, Hep3B cells incubated in LX-2 CM showed lower apoptosis rate than that in normal medium. (C) Western blot analysis of active caspase 8 and 3, GAPDH as internal control. Cisplatin induced caspase 8 and 3 activation products were profoundly reduced when it collaborated with LX-2 CM exposure. (D) HSCs modulate chemoresistance of HCC in vivo. Hep3B cells were subcutaneously injected or co-injected with LX-2 cells into nude mice to form xenografts. In response to cisplatin treatment, tumors from mice of co-injection with Hep3B cells and LX-2 cells were larger and heavier than that of single injection with Hep3B cells. *p < 0.05, **p < 0.01, ***p < 0.001, mean ± SEM.

In the first place, we sought to investigate whether HSCs affected the cisplatin resistance on hepatoma cells in vitro. We used human HSC line LX-2 and chemotherapeutic agent cisplatin to investigate their effects on human HCC cell line Hep3B. To examine the cytotoxic effect of cisplatin, Hep3B cells with or without LX-2 CM exposure were treated with 10µg/ml of cisplatin and cell viability was analyzed. Cisplatin caused cell proliferation arrest, corresponding to a decreased percentage of cell viability. Interestingly, Hep3B cells with LX-2 CM exposure displayed significantly attenuated cytotoxicity in response to cisplatin (Figure 1A). This unexpected sensitization to cytotoxicity was further confirmed by measurement of apoptosis using flow cytometry with Annexin V-FITC and PI double staining (Figure 1B).

Propagation of the apoptotic signal occurs through hierarchical proteolytic cleavage of a class of cysteine proteases with aspartic acid specificity, the caspases. Caspase 8 is considered as activator caspase to ignite death machine and caspase 3 has been reported to act as apoptotic executioner [12]. In our study, autocatalytic activation of caspase 8 and 3 was found in Hep3B cells as the result of cisplatin treatment. Consistently, cisplatin induced caspase 8 and 3 activation products were profoundly reduced when it collaborated with LX-2 CM exposure (Figure 1C).

We also determined whether HSCs modulated hepatoma cells chemoresistance in vivo. We adopted subcutaneous tumorigenicity experiments in null mice as HCC model to evaluate the effects of HSCs in cisplatin resistance. Suspension of Hep3B cells or mixtures of Hep3B cells and LX-2 cells were injected subcutaneously into nude mice to form xenografts and their responses to cisplatin were estimated by tumor volume and mass weigh. Cispltin induced growth arrest was evident, particularly in the tumor formation of Hep3B cells single injection. Interestingly, the growth inhibition of cisplatin was partly mitigated by LX-2 cells (Figure 1D). Taken together, these experiments confirmed that growth inhibitory and apoptosis effect of cisplatin on hematoma cell could be efficiently inhibited by HSCs.

### Hep3B cells upon LX-2 CM exposure exhibit molecular changes consistent with EMT

**Figure 2:**
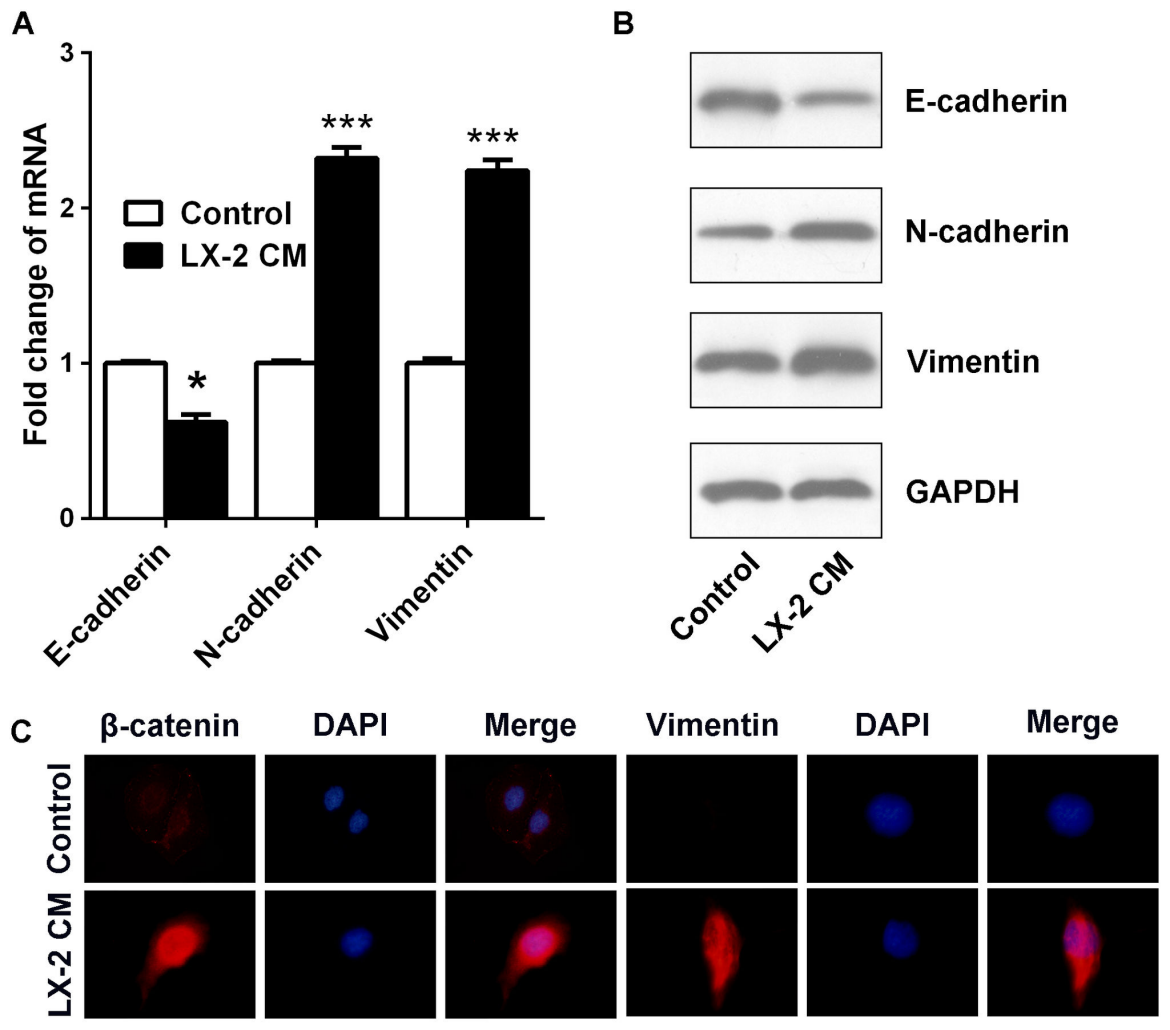
Hep3B cells upon LX-2 CM exposure exhibit molecular changes which are consistent with EMT. (A) Real-time PCR analysis of E-cadherin, N-cadherin and Vimentin mRNA levels and (B) Protein expressions of E-cadherin, N-cadherin and Vimentin in Hep3B cells were analyzed by Western blotting, GAPDH as endogenous control. E-cadheirn expression was suppressed, while Vimentin and N-cadherin expressions were increased in Hep3B cells with LX-2 CM exposure. (C) Immunofluorescence analysis of β-catenin and Vimentin expressions. After LX-2 CM exposure, β-catenin was nuclear translocation and Vimentin was induced in Hep3B cells. *p < 0.05, ***p < 0.001, mean ± SEM.

Emerging evidences associate chemoresistance with acquisition of EMT in cancer. To determine if specific molecular changes consistent with EMT, we examined the expressions of epithelial and mesenchymal markers. We found a profound reduction of E-cadherin expression and pronounced upregulation of N-cadherin and Vimentin in Hep3B cells in reply to LX-2 CM exposure (Figure 2A and B). In addition, immunofluorescence staining showed that β-catenin was localized and organized in the membranes of Hep3B cells incubated in normal condition. In contrast, upon LX-2 CM exposure, β-catenin was distributed largely in the cell nucleus. The results were consistent with previous studies that alteration of the E-Cadherin/β-Catenin complex and nuclear translocation of β-Catenin drive the development of the mesenchymal phenotypes [13]. Furthermore, immunostaining results also indicated that Vimentin was induced in the cytoplasm of Hep3B cells upon LX-2 CM exposure (Figure 2C).

### LX: 2 CM upregulate CSC-like properties of Hep3B cells

**Figure 3:**
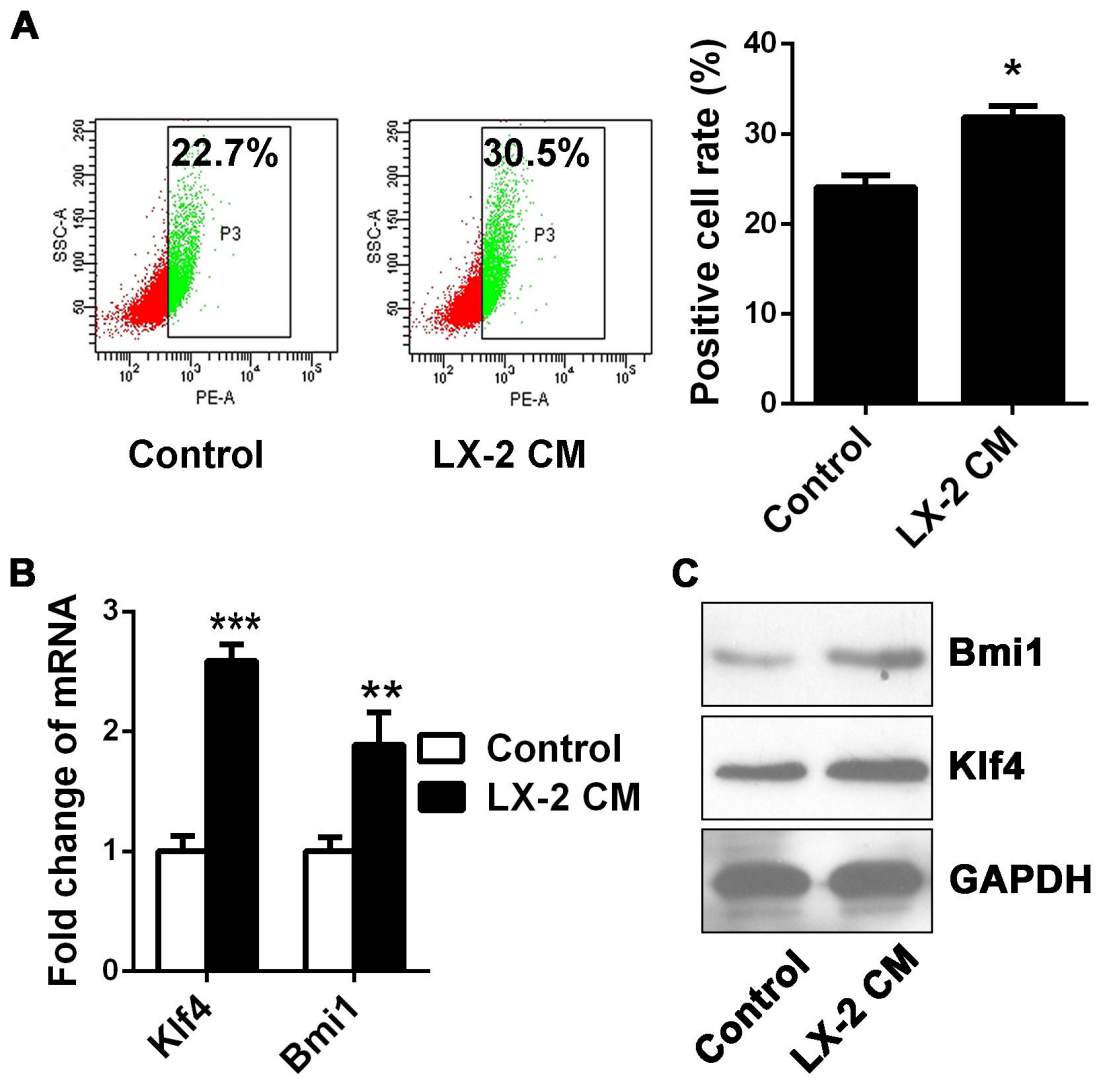
Upregulation of CSC-like properties in Hep3B cells with LX-2 CM. (A) Flow cytometry analysis the CSC surface marker CD133. The percentage of CD133 positive cells was increased in Hep3B cells upon LX-2 CM exposure. (B) Real-time PCR and (C) Western blot analysis of CSC transcriptional factors Bmi1 and Klf4 mRNA expressions in Hep3B cells. Both Bmi1 and Klf4 expressions were upregulated in Hep3B cells after LX-2 CM exposure. *p < 0.05, **p < 0.01, mean ± SEM.

As key tumor-initiating cells, CSCs may play an integral role in recurrence following chemotherapy. The mechanisms involved in chemoresistance of CSCs are complex, including overexpression of ABC transporters, detoxification enzymes (aldehyde dehydrogenase), low cell turn over rate and activation of the DNA check point response [14]. To assess if some of CSC properties were conferred by LX-2 CM, we performed flow cytometry to assay for the presence of CD133, a conserved cell surface molecule that has been identified as a marker of cancer stem-like cells in Hep3B cells. The results indicated that the CD133 positive cell population was considerably richen in Hep3B cells with LX-2 CM exposure (Figure 3A). We further ascertained the induction of stem-like cell traits by detection of two CSC transcriptional factors Bmi1 and Klf4, both of which have been proved to play an essential role in sustaining self-renewing cell activity. The increase in Bmi1 and Klf4 levels in Hep3B cells with LX-2 CM exposure was confirmed by real-time PCR and Western blotting analysis (Figure 3B and C). These data revealed that LX-2 CM exposure not only triggered EMT, but also in turn transited Hep3B cells into the stem like state.

### LX: 2 cells secrete HGF into CM

**Figure 4:**
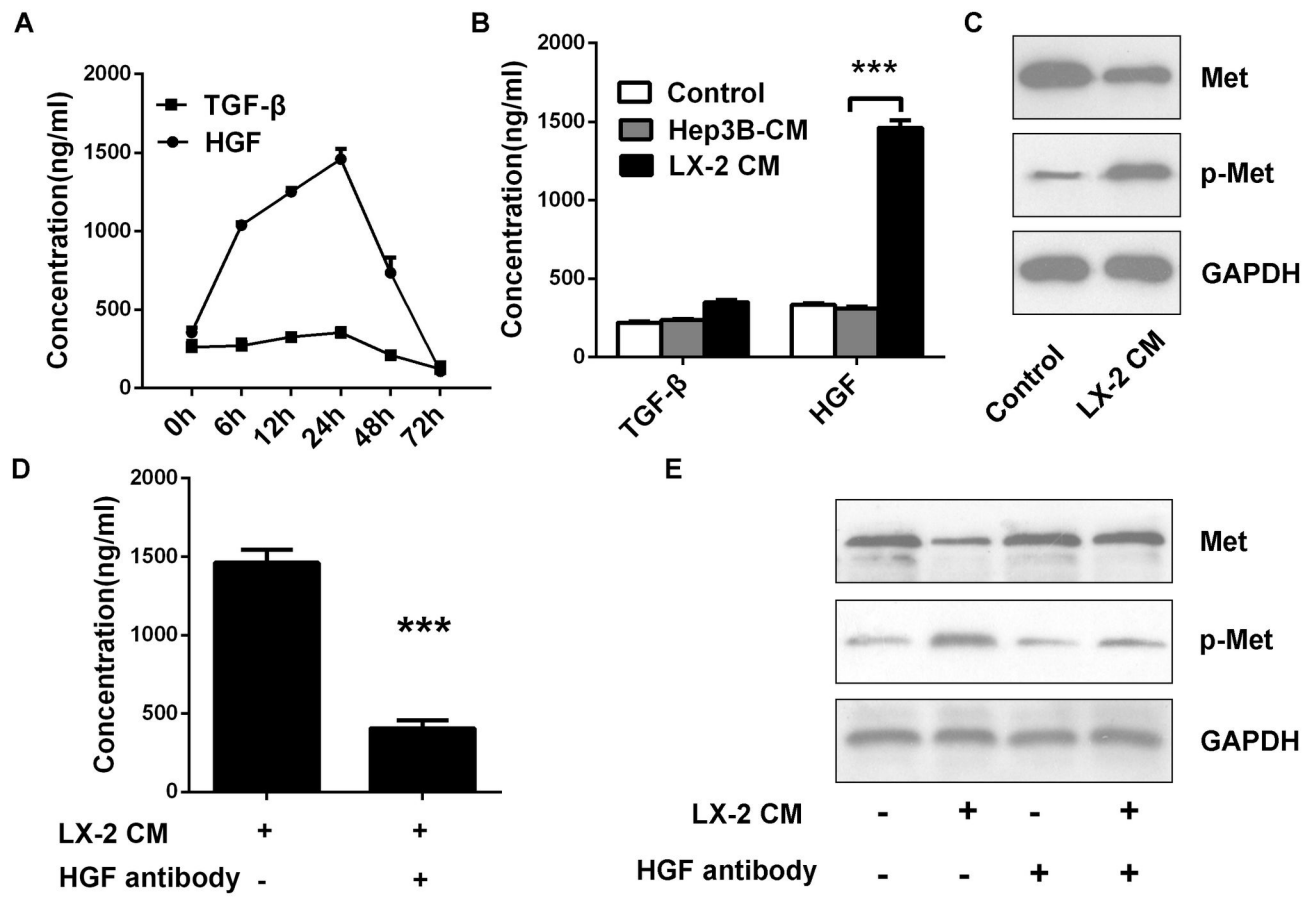
LX 2 cells secrete HGF into CM. (A) The time course analysis of HGF and TGF-β levels in LX-2 CM. The level of HGF was dramatically increased in the first 24h in medium cultured LX-2 cells, while TGF-β remained low level. After 24h, both levels were decreased might be due to nutrient depletion and other reasons. In our study, LX-2 CM referred to medium incubated LX-2 cells for 24h. (B) Analysis of HGF level in normal medium, Hep3B CM and LX-2 CM. HGF level of LX-2 CM was particularly higher than other two solvents. (C) Western blot analysis of Met and p-Met level in Hep3B cells. In the role of LX-2 CM, Met protein level was reduced and p-Met protein level was increased in Hep3B cells. (D) HGF neutralizing antibody could effectively reduce the HGF level of LX-2 CM. (E) Depletion of HGF in LX-2 CM block its effect on activation of Met. P-Met was at basic level in Hep3B cells upon HGF immunodepleted LX-2 CM exposure. ***p < 0.001, mean ± SEM.

HSCs synthesize and secrete multiplex of cytokines, growth factors and chemokines to create tumor microenvironment. To understand the underlying mechanism by which LX-2 CM promoted the EMT and CSC phenotypes of Hep3B cells, we collected the CM from the LX-2 cells cultured in 2D culture. We chose to screen for two cytokines in tumor environment-namely, HGF and TGF-β which have been previously identified as potent EMT inducers. Interestingly, the results indicated that HGF instead of TGF-β was secreted into CM by LX-2 cells (Figure 4A). We next measured HGF secretion from the HCC line Hep3B and found HGF level in cultured media of Hep3B cells was quite minimal (Figure 4B). In our study, LX-2 CM referred media to 24h cultured LX-2 cells.

In order to further verify that LX-2 cells secreted HGF into CM, we also interrogated whether there was a change of HGF/Met signaling pathway in Hep3B cells upon LX-2 CM exposure. It was not surprising that an increase in Met phosphorylated at tyrosine residues 1234/1235 (p-Met) was evident in Hep3B cells upon LX-2 CM exposure (Figure 4C). The HGF level in LX-2 CM could effectively be reduced by using HGF neutralizing antibody (Figure 4D). And p-Met at the minimum and elementary level was found in Hep3B cells upon HGF immunodepleted LX-2 CM exposure (Figure 4E). Collectively, these results suggested that LX-2 cells derived HGF, but not TGF-β promoted the EMT and CSC phenotypes in Hep3B cells, and prompted us to focus on HGF in the subsequent experiments.

### Depletion of HGF in LX-2 CM block its effect on promotion of the EMT, CSC phenotypes and cisplatin resistance

**Figure 5:**
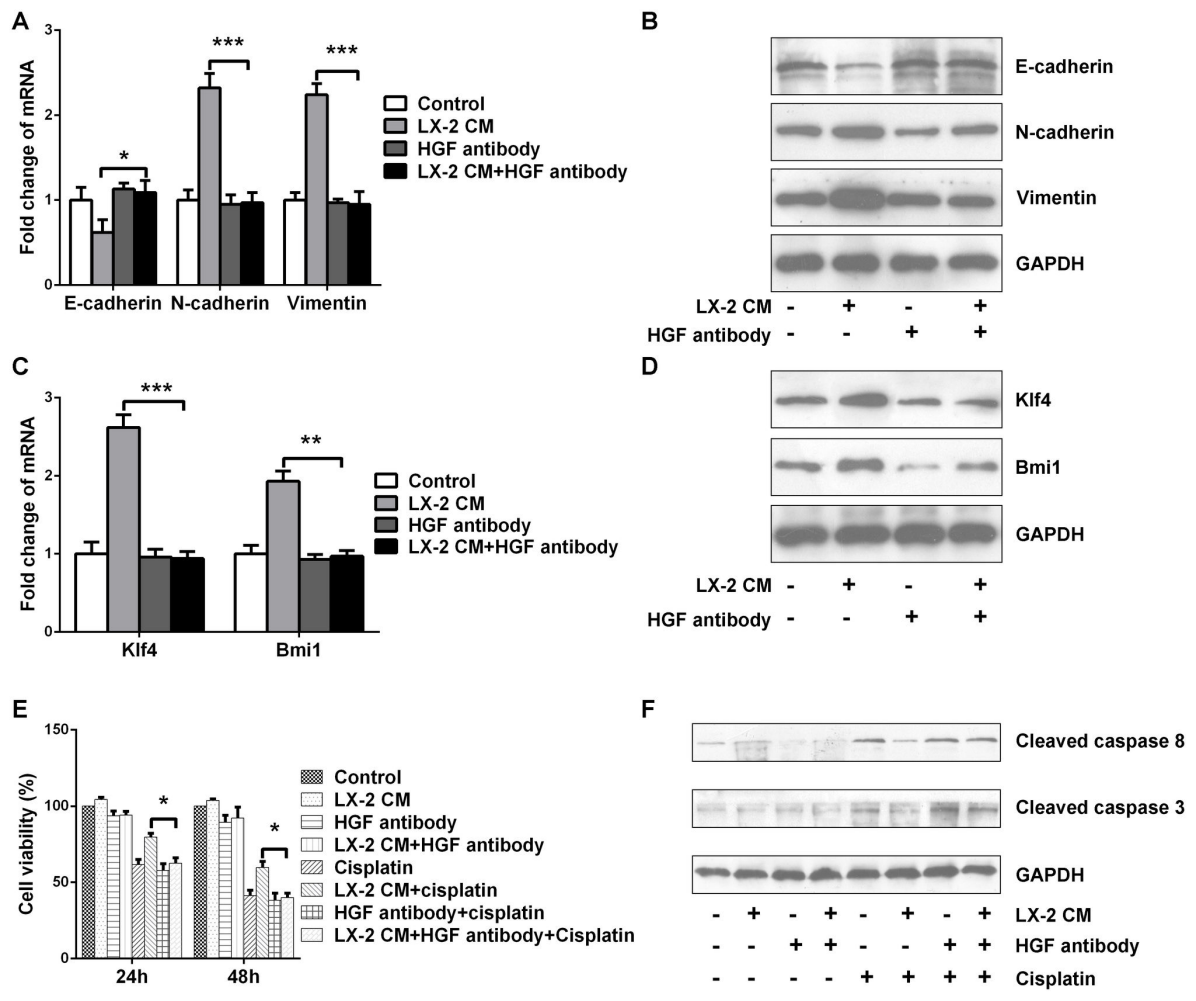
Depletion of HGF in LX-2 CM block its effects on promotion of the EMT, CSC phenotypes and cisplatin resistance. (A) Real-time PCR and (B) Western blot analysis of E-cadherin, N-cadherin and Vimentin expressions. Compared to LX-2 CM exposure, E-cadherin level was higher, while N-cadherin and Vimentin levels were lower in Hep3B cells upon HGF-depleted LX-2 CM exposure. (C) Real-time PCR and (D) Western blot analysis of Bmi1 and Klf4 expressions. Compared to LX-2 CM exposure, Bmi1 and Klf4 expressions were inhibited in Hep3B cells with HGF-depleted LX-2 CM exposure. (E) CCK8 analysis of cell viability. (F) Western blot analysis of active caspase 8 and 3 levels. In response to cisplatin treatment, Hep3B cells upon HGF-depleted LX-2 CM exposure showed lower cell viability and higher active caspase 8 and 3 levels than that upon LX-2 CM exposure. *p < 0.05, **p < 0.01, ***p < 0.001, mean ± SEM.

Having established that LX-2 cells secrete HGF to activate HGF/Met signaling, we sought to determine whether HGF/Met signaling was responsible for the LX-2 CM mediated EMT and CSC traits in Hep3B cells. Previous data had shown that HGF neutralizing antibody could effectively reduce the HGF level of LX-2 CM and activation of HGF / MET signaling was inhibited in Hep3B cells upon HGF immunodepleted LX-2 CM exposure (Figure 4). Therefore, we used HGF antibody as an immunoprecipitation-based approach to deplete HGF in LX-2 CM to determine whether HGF was responsible for promoting the EMT, CSC features and cisplatin resistance in Hep3B cells. Following depletion of HGF in LX-2 CM, the LX-2 CM was no longer able to reduce epithelial marker E-cadherin expression and upregulate mesenchymal markers N-cadherin and Vimentin expressions (Figure 5A and B). Consistently, compared to LX-2 CM exposure, CSC transcriptional factors Bmi1 and Klf4 levels were decreased in Hep3B cells incubated with HGF-depleted LX-2 CM (Figure 5C and D). Meanwhile, in response to cisplatin treatment, Hep3B cells with HGF-depleted LX-2 CM exposure showed lower cell viability and higher active caspase 8 and 3 than that of LX-2 CM exposure(Figure 5E and F). These data indicated that the HGF that we identified was the key molecule in LX-2 CM to promote the EMT, CSC phenotypes and cisplatin resistance in Hep3B cells.

## Discussion

Since chemotherapy drugs were widely used in clinical practice, the phenomenon of chemoresistance has become a major obstacle in tumor chemotherapy [15]. Therefore, a thorough understanding of the mechanisms by which the residual tumor cells survive chemotherapy treatment is essential to find out more effective chemotherapeutic strategies. Recent studies have proposed that both genetic alterations of cancer cells and their surrounding microenvironment could be associated with chemoresistance [16]. Stromal cells could potentially induce chemoresistance acquisition in tumor cells, including cell-cell and cell-matrix interactions, local release of soluble factors and so on [17]. In HCC, except for paracancerous location, HSCs have abilities to infiltrate tumor stroma and secrete multiple of extracellular matrix, cytokines and growth factors in the tumor environment [4]. Previous studies have confirmed that HSCs participate in regulating tumorigenesis, invasion and migration of HCC. In this study, we aimed to investigate the molecular mechanism of the effects of HSCs in cisplatin resistance of HCC and associated cellular behaviors.

By using HSC cell line LX-2 and chemotherapy drug cisplatin, we demonstrate that HSCs play a functional role in chemoresistance of HCC presumably through their secreted HGF in tumor milieu to induce EMT and upregulate CSC-like traits of HCC via HGF/Met signaling. This was confirmed by (1) cisplatin resistance of Hep3B cells was enhanced upon LX-2 CM exposure in vitro as well as co-injected with LX-2 cells in null mice(2); Hep3B cells in presence of LX-2 CM underwent EMT and upregulation of CSC-like properties; (3) LX-2 cells produced and secreted HGF in CM; (4) HGF receptor tyrosine kinase Met of Hep3B cells was activated upon LX-2 CM exposure; (5) HGF neutralizing antibody could effectively reduce the HGF level of LX-2 CM and activation of HGF / MET signaling was inhibited in Hep3B cells upon HGF immunodepleted LX-2 CM exposure; (6) depletion of HGF in LX-2 CM also blocked its effect on promotion of the EMT, CSC-like features as well as cisplatin resistance in Hep3B cells. Our finding that cisplatin resistance of Hep3B cells was enhanced upon LX-2 CM exposure in vitro as well as co-injection with LX-2 cells in null mice show that HSCs involve in HCC chemoresistance.

In agreement with previous findings that induction of EMT acquired resistance to cancer chemotherapy [18]. Hep3B cells cultured in presence of LX-2 CM underwent EMT, in which process polarized epithelial cells are converted into motile cells with loss of the epithelial phenotypes (degradation of E-cadherin/β-catenin complex), acquirement of mesenchymal phenotypes (upregulation of N-cadherin and Vimentin) and increase of specific transcriptional factors (Snail family members) [19]. In our study, the expression of E-cadherin was decreased and β-catenin was translocated in the nucleus of Hep3B cells after LX-2 CM exposure. E-cadherin/β-catenin complex is related to adherens junctions and cell-cell adhesion, one of the cardinal features of epithelial cells [20]. Key targets of the pathways that induce EMT include the adherens junction components E-cadherin and β-catenin [21]. In pace with degradation of E-cadherin, β-catenin was nuclear translocation acting as a transcriptional regulator and the principal effector of the Wnt pathway [22]. Aberrant nuclear expression of β-catenin confers tumor cell two decisive abilities: EMT and stem cell formation [23]. In addition, non-epithelial cadherin, N-cadherin were found to induce a mesenchymal-scattered phenotype and Vimentin is a type III intermediate filament protein that is normally found in mesenchymal cells [24,25]. They are both upregulated in Hep3B cells incubated with LX-2 CM. CSCs are a heterogeneous population of tumor cells with unique biological characteristics, including self-renewal capability, stem cell signaling pathways, relative quiescence and resistance to standard chemotherapy and radiotherapy [26]. CD133 has been used as a surface marker for the identification and isolation of a putative CSC population from several tumors, including HCC [27]. Furthermore, CD133 positive CSCs in HCC was shown to confer with ability of chemoresistance [28]. Bmi1 and Klf4 are transcriptional factors to maintain self renewal of CSCs [29,30]. In addition, previous studies have shown that Bmi1 and Klf4 are also essential to promote EMT [31,32]. In our study, the levels of CD133, Bmi1 and Klf4 expressions in Hep3B cells were degrees of increase. These results indicate that HSCs promote EMT and CSC traits of HCC.

A variety of extracellular stimuli have the potential to induce EMT. HGF identical to scatter factor (SF) is a glycoprotein expressed ubiquitously in tumor stromal microenvironment, particularly active in the reactive stroma of tumors to promote tumorigenesis, growth and survival [33]. This fascinating cytokine transduces its activities via its receptor Met, coupled to a number of transducers integrating the HGF/Met signal to the cytosol and the nucleus [34]. Upon HGF binding, Met autophosphorylation occurs on tyrosine residues Y1234 and Y1235 within the activation loop of the TK domain, inducing kinase activity, while phosphorylation on Y1349and Y1356 near the carboxyl terminus forms a docking site for intracellular adapters that transmit signals downstream [35]. Actually, aberrant activation of HGF/Met signaling pathway contributes to oncogenesis and tumour progressionin several cancers and promotes aggressive cellular invasiveness [36]. In our study, the high level of HGF in LX-2 CM indicated that HGF might be the key molecule to mediate the interaction between HSCs and HCC. We also demonstrated that LX-2 CM exposure, an increase in Met phosphorylated at tyrosine residues 1234/1235 was manifest in Hep3B cells. It indicated that HGF was indeed secreted in LX-2 CM and HGF/Met pathway was activated in Hep3B cells upon LX-2 CM treatment. Furthermore, depletion of HGF in LX-2 CM blocks its effect on promotion Met activation as well as the EMT, CSC phenotypes and cisplatin resistance. These data prove that HGF is the key molecule to mediate EMT, CSC phenotypes and chemoresistance of HCC via HGF/Met signaling.

In summary, although multiple cytokines and growth factors can promote chemoresistance of HCC, we demonstrate in this study a possible mechanism that secreting HGF into tumor milieu, HSCs decrease hepatoma cells sensitization to chemotherapeutic agents by promotion of the EMT and CSC features via HGF/Met signaling.
